# 

**Published:** 1897-11

**Authors:** 


### CONTENTS.

**ORIGINAL COMMUNICATIONS—**

DIPHTHERIA ANTITOXIN, WITH REPORT OF CASES. By Henry R. Slack, Ph.M., M.D., LaGrange, Ga. .......... 577

CHRONIC NON-SUPPURATIVE OTITIS MEDIA. By S. Mac Cuen Smith, M.D., Philadelphia, Pa .......... 584

CLINICAL NOTES ON OBSTETRIC SURGERY. By Jopeph Eve Allen, M.D., Augusta, Ga. 588

SOME NERVOUS TROUBLES ASSOCIATED WITH DISEASES OF THE EAR. By Professor A. A. Foucher, Montreal, Canada.......... 597

HYSTERIA IN EARLY LIFE. By Augustus A. Eshner, M.D., Philadelphia, Pa.......... 603

**SOCIETY REPORTS—**

ATLANTA SOCIETY OF MEDICINE.......... 612

**EDITORIAL—**

MEDICAL EXPERT TESTIMONY. THE ABUSE AND THE REMEDY.......... 616

SELECTIONS AND ABSTRACTS.......... 621

MEDICAL ITEMS.......... 637

BOOK REVIEWS.......... 643

MEDICAL ITEMS—CONTINUED.......... x, xii, xiv, xvi

ADVERTISERS' INDEX TO ADVERTISEMENTS.......... xviii

CLASSIFIED INDEX TO ADVERTISEMENTS.......... xxxvii

